# Supplementary material for: Roles of Cytokines in the Temporal Changes of Microglial Membrane Currents and Neuronal Excitability and Synaptic Efficacy in ATP-Induced Cortical Injury Model
Source: Int J Mol Sci. 2021 Jun 25;22(13):6853. doi: 10.3390/ijms22136853 (PMC8268462; doi:10.3390/ijms22136853)
Supplement: Supplementary file 1 [file ijms-22-06853-s001.zip › Supplementary Figure S1-CHK.pdf]

**Supplementary Figure S1. Measurement of soma area of CX3CR1-GFP(+) microglia using Image J.**

**A.**

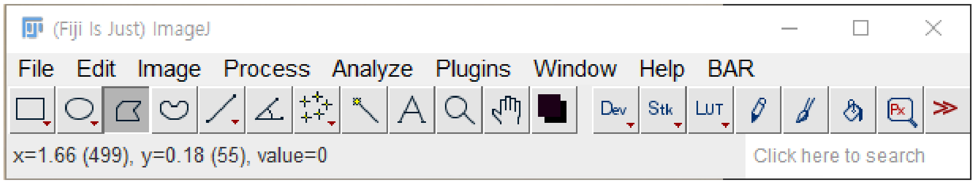


**B.**

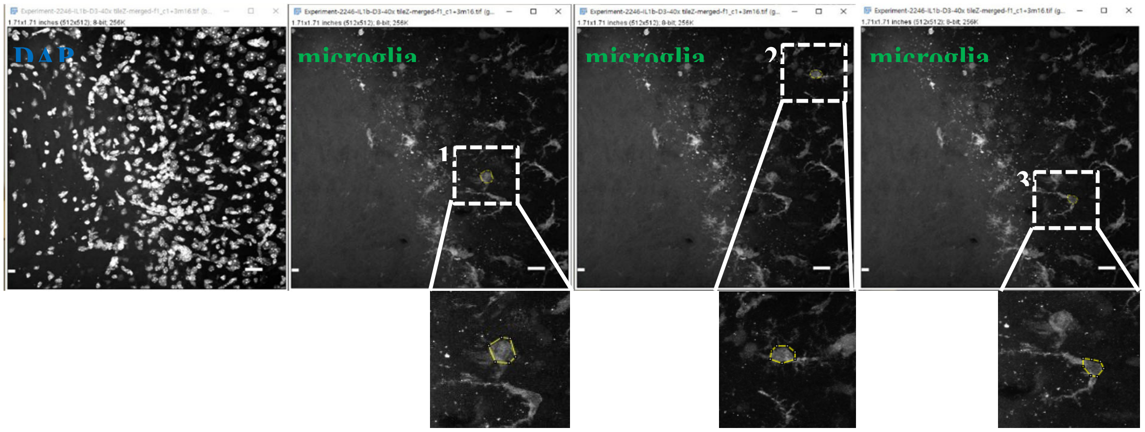


A. Capture of Fiji Image J software.

First, open the 400x image taken with an Olympus confocal microscope with Image J and separate the fluorescent signals to blue (DAPI) and green (microglia), and measure the soma area of the microglia.

B. One example image of 3 dpi brain tissue sample.

*Far left*, DAPI signal image representing the nucleus of cells (Far left). Next images present three example microglia (GFP, dotted squares, 1-3) of the same sample for measuring the soma

area with polygon selection method. *Scale bar*, 20 μm. Below are the expanded images of the dotted squares. The dotted line indicates the soma region of a microglia for the analysis.
