# Supplementary material for: Roles of Cytokines in the Temporal Changes of Microglial Membrane Currents and Neuronal Excitability and Synaptic Efficacy in ATP-Induced Cortical Injury Model
Source: Int J Mol Sci. 2021 Jun 25;22(13):6853. doi: 10.3390/ijms22136853 (PMC8268462; doi:10.3390/ijms22136853)
Supplement: Supplementary file 1 [file ijms-22-06853-s001.zip › Supplementary File-Table S1-CHK.pdf]

Table S1. Summary of statistics for Figures 1, 3, 4, 5, 6, and 7.

Figures		Variable	statistics	N	F value	P value	Post hoc test
**Figure 1**	**C**	Core + Penumbra area of 3 dpi	Two-tailed t-test	7 dpi: 8<br>28 dpi: 4<br>Nd 3: 7<br>Nd 7: 4<br>Nd 28: 5	F=0.15<br>F=0.47<br>F=0.63<br>F=0.12<br>F=0.48	P < 0.001<br>P < 0.001<br>P < 0.001<br>P < 0.001<br>P < 0.001	-
	**D**	Soma size of con	Two-tailed t-test	3 dpi: 38<br>7 dpi: 38<br>28 dpi: 38<br>Nd 3: 31<br>Nd 7: 29<br>Nd 28: 24	F < 0.05<br>F < 0.05<br>F=0.62<br>F < 0.05<br>F < 0.05<br>F=0.1	P < 0.001<br>P < 0.001<br>P=0.69<br>P < 0.001<br>P < 0.001<br>P=0.99	-
	**E**	GFP F. I. of con	Two-tailed t-test	3 dpi: 13<br>7 dpi: 13<br>28 dpi: 4<br>Nd 3: 7<br>Nd 7: 4<br>Nd 28: 4	F < 0.05<br>F=0.09<br>F=0.2<br>F=0.21<br>F < 0.05<br>F=0.67	P < 0.001<br>P < 0.001<br>P < 0.001<br>P=0.001<br>P=0.17<br>P=0.28	-
	**F**	DAPI num. of con	Two-tailed t-test	3 dpi: 13<br>7 dpi: 13<br>28 dpi: 4<br>Nd 3: 7<br>Nd 7: 4<br>Nd 28: 4	F=0.05<br>F=0.63<br>F=0.92<br>F=0.89<br>F=0.49<br>F=0.5	P=0.75<br>P < 0.001<br>P < 0.001<br>P=0.84<br>P=0.26<br>P=0.45	-
**Figure 3**	**A**	Num. of spikes	Two-way ANOVA	3 dpi: 17<br>7 dpi: 13<br>28 dpi: 11<br>Nd 3: 16<br>Nd 7: 9<br>Nd 28: 14	65.83	P < 0.001	Tukey's multiple comparison
	**B**	Vm	Two-tailed t-test		F=0.2<br>F=0.68<br>F=0.3<br>F=0.34<br>F=0.72<br>F=0.18	P=0.71<br>P=0.56<br>P=0.89<br>P=0.78<br>P=0.51<br>P=0.64	-
	**C**	I/V	Two-way ANOVA		11.98	P < 0.001	Tukey's multiple comparison
	**D**	Rheobase of con	Two-tailed t-test		3 dpi: 0.18<br>7 dpi: 0.41<br>28 dpi: F < 0.05<br>Nd 3: F < 0.05<br>Nd 7: 0.34<br>Nd 28: 0.43	P=0.001<br>P=0.19<br>P=0.06<br>P=0.41<br>P=0.17<br>P=0.003	-
	**E**	Rm of con	Two-tailed t-test		3 dpi: F < 0.05<br>7 dpi: 0.19<br>28 dpi: F < 0.05<br>Nd 3: 0.2<br>Nd 7: F < 0.05<br>Nd 28: 0.4	P=0.07<br>P=0.67<br>P=0.99<br>P=0.74<br>P=0.03<br>P=0.03	-
	**F**	Cm	Two-tailed t-test		3 dpi: 0.59<br>7 dpi: 0.98<br>28 dpi: F < 0.05<br>Nd 3: 0.61<br>Nd 7: F < 0.05<br>Nd 28: 0.33	P=0.002<br>P=0.02<br>P=0.33<br>P=0.02<br>P < 0.001<br>P=0.006	-
	**G**	Peak amp. 30	Two-tailed t-		3 dpi: F < 0.05<br>7 dpi: 0.06	P=0.22<br>P=0.38	-

		pA	test		28 dpi: F < 0.05	P=0.25	
		90 pA			3 dpi: 0.1<br>7 dpi: F < 0.05<br>28 dpi: 0.4	P=0.17<br>P=0.02<br>P=0.06	
		150 pA			3 dpi: 0.16<br>7 dpi: 0.06<br>28 dpi: 0.43	P=0.17<br>P=0.03<br>P=0.09	
	**H**	Half-width 30 pA	Two-tailed t-test		3 dpi: F < 0.05<br>7 dpi: F < 0.05<br>28 dpi: 0.41	P=0.26<br>P=0.15<br>P=0.39	-
		90 pA			3 dpi: 0.23<br>7 dpi: 0.99<br>28 dpi: 0.52	P=0.78<br>P=0.53<br>P=0.85	
		150 pA			3 dpi: F < 0.05<br>7 dpi: F < 0.05<br>28 dpi: F < 0.05	P=0.71<br>P=0.06<br>P=0.49	
	**J**	Freq. of sIPSC Con	Two-tailed t-test	Con: 14<br>3 dpi: 16<br>7 dpi: 16<br>28 dpi: 14	3 dpi: F < 0.05<br>7 dpi: 0.86<br>28 dpi: 0.45	P=0.04<br>P=0.45<br>P=0.28	-
		3 dpi			7 dpi: F < 0.05<br>28 dpi: 0.06	P=0.003<br>P=0.34	
	**K**	Peak amp. of sIPSC Con	Two-tailed t-test		3 dpi: 0.21<br>7 dpi: 0.51<br>28 dpi: 0.81	P=0.02<br>P=0.195<br>P=0.89	-
		3 dpi			7 dpi: 0.54<br>28 dpi: 0.32	P=0.24<br>P=0.02	
	**L**	Freq. of sEPSC Con	Two-tailed t-test		3 dpi: 0.06<br>7 dpi: 0.57<br>28 dpi: 0.11	P=0.01<br>P=0.3<br>P=0.93	-
	**M**	Peak amp. of sEPSC	Two-tailed t-test		3 dpi: F < 0.05<br>7 dpi: F < 0.05<br>28 dpi: 0.69	P=0.5<br>P=0.66<br>P=0.09	-
	**N**	E/I amp. ratio	Two-tailed t-test		3 dpi: F < 0.05<br>7 dpi: 0.84<br>28 dpi: F < 0.05	P=0.18<br>P=0.44<br>P=0.16	-
	**O**	E/I freq. ratio Con	Two-tailed t-test		3 dpi: F < 0.05<br>7 dpi: F < 0.05<br>28 dpi: 0.54	P < 0.001<br>P=0.47<br>P=0.14	-
**Figure 4**	**A**	Current density	Two-way ANOVA	Con: 28<br>3 dpi: 30<br>7 dpi: 15<br>28 dpi: 15<br>Nd 3: 11<br>Nd 7: 11	175.4	P < 0.001	Tukey's multiple comparison
		-150 mV con	Two-tailed t-test		3 dpi: F < 0.05<br>7 dpi: F < 0.05<br>28 dpi: 0.64<br>Nd 3: 0.24<br>Nd 7: 0.64	P < 0.001<br>P < 0.001<br>P=0.001<br>P=0.78<br>P=008	-
		-50 mV con			3 dpi: F < 0.05<br>7 dpi: 0.32<br>28 dpi: F < 0.05<br>Nd 3: 0.89<br>Nd 7: 0.21	P < 0.001<br>P < 0.001<br>P=0.02<br>P=0.35<br>P=0.49	
		50 mV con			3 dpi: F < 0.05<br>7 dpi: 0.13<br>28 dpi: 0.17<br>Nd 3: F < 0.05<br>Nd 7: 0.06	P < 0.001<br>P=0.02<br>P=0.25<br>P < 0.001<br>P=0.21	
	**B**	Vm	Two-		3 dpi: 0.769	P=0.09	-

		Con	tailed t-<br>test		7 dpi: 0.2<br>28 dpi: 0.38<br>Nd 3: 0.49<br>Nd 7: 0.29	P=0.39<br>P=0.003<br>P=0.68<br>P=0.39	
	**C**	Vr<br>Con	Two-<br>tailed t-<br>test		3 dpi: F < 0.05<br>7 dpi: F < 0.05<br>28 dpi: F < 0.05<br>Nd 3: 0.06<br>Nd 7: F < 0.05	P < 0.001<br>P < 0.001<br>P=0.001<br>P=0.61<br>P=0.21	
	**D**	Cm<br>Con	Two-<br>tailed t-<br>test		3 dpi: F < 0.05<br>7 dpi: 0.9<br>28 dpi: 0.56<br>Nd 3: 0.52<br>Nd 7: 0.41	P=0.03<br>P=0.72<br>P=0.93<br>P=0.32<br>P=0.98	-
	**E**	Rm<br>Con	Two-<br>tailed t-<br>test		3 dpi: F < 0.05<br>7 dpi: F < 0.05<br>28 dpi: 0.38<br>Nd 3: 0.49<br>Nd 7: F < 0.05	P < 0.001<br>P < 0.001<br>P < 0.001<br>P=0.48<br>P=0.49	-
	**N**	-150 mV<br>con	Two-<br>tailed t-<br>test	Cs: 5<br>Ba: 10<br>4AP: 9<br>Qui.: 15<br>ML133: 16<br>Pax.: 10<br>A740.: 11<br>Sur.: 12	Cs: 0.35<br>Ba: 0.05<br>4AP: 0.45<br>Qui.: 0.44<br>ML133: 0.1<br>Pax.: 0.14<br>A740.: 0.92<br>Sur.: 0.99	P=0.55<br>P=0.55<br>P < 0.001<br>P=0.89<br>P=0.04<br>P=0.02<br>P=0.001<br>P < 0.001	-
		-150 mV<br>3 dpi			Cs: F < 0.05<br>Qui.: F < 0.05<br>4AP: F < 0.05<br>Ba: F < 0.05<br>ML133: F < 0.05<br>Pax.: F < 0.05<br>A740.: F < 0.05<br>Sur.: F < 0.05	P < 0.001<br>P < 0.001<br>P=0.003<br>P < 0.001<br>P < 0.001<br>P=0.01<br>P < 0.001<br>P=0.003	
		-50 mV<br>Con			Cs: 0.91<br>Ba: F < 0.05<br>4AP: 0.09<br>Qui.: 0.99<br>ML133: 0.3<br>Pax.: 0.55<br>A740.: F < 0.05<br>Sur.: 0.69	P=0.17<br>P=0.02<br>P=0.005<br>P=0.25<br>P=0.09<br>P=0.63<br>P=0.78<br>P=0.02	
		-50 mV<br>3 dpi			Cs: F < 0.05<br>Ba: 0.54<br>4AP: 0.15<br>Qui.: F < 0.05<br>ML133: F < 0.05<br>Pax.: F < 0.05<br>A740.: F < 0.05<br>Sur.: F < 0.05	P < 0.001<br>P=0.67<br>P=0.07<br>P < 0.001<br>P < 0.001<br>P=0.001<br>P < 0.001<br>P=0.003	
		50 mV<br>Con			Cs: 0.19<br>Ba: F < 0.05<br>4AP: F < 0.05<br>Qui.: F < 0.05<br>ML133: F < 0.05<br>Pax.: F < 0.05<br>A740.: F < 0.05<br>Sur.: F < 0.05	P=0.11<br>P < 0.001<br>P=0.007<br>P=0.84<br>P < 0.001<br>P=0.01<br>P < 0.001<br>P < 0.001	

		50 mV<br>3 dpi			Cs: F < 0.05<br>Qui.: F < 0.05<br>4AP: F < 0.05<br>Ba: F < 0.05<br>ML133: F < 0.05<br>Pax.: 0.08<br>A740.: F < 0.05<br>Sur.: F < 0.05	P < 0.001<br>P < 0.001<br>P < 0.001<br>P < 0.001<br>P < 0.001<br>P < 0.001<br>P < 0.001<br>P < 0.001	
**Figure 5**	**A**	Num. of spikes	Two-way ANOVA	Con: 29<br>3 dpi: 16<br>TNFα: 11<br>IL1β: 11<br>IL10: 10<br>IL4: 16<br>ATP(1mM): 14<br>ATPγS (100μM): 6	102.8	P < 0.001	Tukey's multiple comparison
	**B**	I/V	Two-way ANOVA		47.63	P < 0.001	Tukey's multiple comparison
	**C**	Num. of spikes	Two-way ANOVA	Con: 29<br>3 dpi: 16<br>3 dpi + IL10: 7<br>3 dpi + IL4: 11	122.1	P < 0.001	Tukey's multiple comparison
	**D**	I/V	Two-way ANOVA		66.09	P < 0.001	Tukey's multiple comparison
**Figure 6**	**A**	Current density	Two-way ANOVA	TNFα: 8<br>IL1β7<br>IL10: 7<br>IL4: 9<br>ATPγS (100μM): 7<br>ATPγS (300μM): 14	15.97	P < 0.001	Tukey's multiple comparison
		-150 mV			TNFα: 0.57<br>IL1β: F < 0.05<br>IL10: 0.16<br>IL4: 0.81<br>ATPγS (100μM): F < 0.05<br>ATPγS (300μM): 0.13	P=0.17<br>P=0.09<br>P=0.81<br>P=0.12<br>P=0.003<br>P=0.39	-
		-50 mV			TNFα: 0.34<br>IL1β: 0.64<br>IL10: F < 0.05<br>IL4: 0.4<br>ATPγS (100μM): F < 0.05<br>ATPγS (300μM): 0.34	P=0.01<br>P=0.69<br>P=0.003<br>P=0.36<br>P=0.05<br>P=0.72	
		50 mV			TNFα: 0.66<br>IL1β: 0.3<br>IL10: F < 0.05<br>IL4: 0.61<br>ATPγS (100μM): 0.97<br>ATPγS (300μM): F < 0.05	P=0.78<br>P=0.24<br>P=0.003<br>P=0.31<br>P=0.91<br>P=0.14	
	**B**	Current density	Two-way ANOVA	TNFα: 9<br>IL1β: 11<br>IL10: 8<br>IL4: 11<br>ATP: 14	16.5	P < 0.001	Tukey's multiple comparison
		-150 mV			TNFα: 0.51<br>IL1β: 0.96<br>IL10: F < 0.05<br>IL4: 0.56<br>ATP: F < 0.05	P=0.04<br>P=0.59<br>P=0.01<br>P=0.4<br>P=0.003	-
		-50 mV			TNFα: 0.55<br>IL1β: 0.4	P=0.23<br>P=0.69	

					IL10: 0.12<br>IL4: 0.11<br>ATP: 0.32	P=0.88<br>P=0.11<br>P=0.69	
		50 mV			TNFα: 0.79<br>IL1β: 0.08<br>IL10: F < 0.05<br>IL4: 1<br>ATP: 0.14	P=0.03<br>P=0.59<br>P=0.08<br>P=0.27<br>P=0.22	
	**C**	Current density	Two-way ANOVA	IL1β+TNFα: 12<br>IL4+IL10: 12<br>IL1β+TNFα +IL4+IL10: 11	11.08	P<0.001	Tukey's multiple comparison
		-150 mV			IL1β+TNFα: 0.52<br>IL4+IL10: 0.4<br>IL1β+TNFα+IL4+IL10 : 0.08	P=0.22<br>P=0.73<br>P=0.7	-
		-50 mV			IL1β+TNFα: 0.89<br>IL4+IL10: 0.56<br>IL1β+TNFα+IL4+IL10 : 0.12	P=0.67<br>P=0.49<br>P=0.78	
		50 mV			IL1β+TNFα: 0.51<br>IL4+IL10: 0.08<br>IL1β+TNFα+IL4+IL10 : 0.21	P=0.39<br>P=0.44<br>P=0.62	
	**D**	Current density	Two-way ANOVA	3 dpi+IL4: 14<br>3 dpi+IL10: 8<br>3dpi+IL4+IL 10: 12	95.74	P<0.001	Tukey's multiple comparison
		-150 mV<br>3 dpi			3 dpi+IL4: 0.09<br>3 dpi+IL10: 0.07<br>3 dpi+IL4+IL10: 0.67	P =0.53<br>P=0.009<br>P=0.05	-
		-50 mV<br>3 dpi			3 dpi+IL4: F < 0.05<br>3 dpi+IL10: 0.72<br>3 dpi+IL4+IL10: 0.61	P =0.09<br>P=0.16<br>P=0.17	
		50 mV<br>3 dpi			3 dpi+IL4: 0.42<br>3 dpi+IL10: 0.07<br>3 dpi+IL4+IL10: F < 0.05	P =0.06<br>P=0.003<br>P =0.06	
**Figure 7**	**A**	Num. of spikes	Two-way ANOVA	Con: 29<br>3 dpi: 16<br>3 dpi+sur.: 12<br>3 dpi+4AP: 11<br>TNFα: 11<br>TNFα+sur.: 10<br>TNFα+4AP: 10	100.2	P < 0.001	Tukey's multiple comparison
	**B**	I/V	Two-way ANOVA		12.02	P < 0.001	Tukey's multiple comparison
	**C**	Vm<br>con	Two-tailed t-test		3 dpi+sur.: 0.46<br>3 dpi+4AP: 0.74<br>TNFα+sur.: 0.85<br>TNFα+4AP: 0.2	P < 0.001<br>P < 0.001<br>P=0.54<br>P=0.009	-
	**D**	Rheobase<br>con	Two-tailed t-test		3 dpi+sur.: F < 0.05<br>3 dpi+4AP: F < 0.05<br>TNFα+sur.: F < 0.05<br>TNFα+4AP: 0.34	P < 0.001<br>P=0.1<br>P=0.88<br>P=0.09	-
	**E**	Cm<br>con	Two-tailed t-test		3 dpi+sur.: 0.31<br>3 dpi+4AP: 0.86<br>TNFα+sur.: 0.31<br>TNFα+4AP: 0.13	P=0.17<br>P=0.89<br>P=0.74<br>P=0.006	-
	**F**	Rm<br>Con	Two-tailed t-test		3 dpi+sur.: F < 0.05<br>3 dpi+4AP: F < 0.05<br>TNFα+sur.: 0.24<br>TNFα+4AP: 0.28	P=0.53<br>P=0.002<br>P=0.02<br>P=0.01	-

Data are mean ± SEM. Asterisks (*) mark significance compared to control group and hashtags (#) mark significance compared to 3 dpi group. */# $p < 0.05$, **/## $p < 0.01$, ***/### $p < 0.001$.
