# Supplementary material for: Roles of Cytokines in the Temporal Changes of Microglial Membrane Currents and Neuronal Excitability and Synaptic Efficacy in ATP-Induced Cortical Injury Model
Source: Int J Mol Sci. 2021 Jun 25;22(13):6853. doi: 10.3390/ijms22136853 (PMC8268462; doi:10.3390/ijms22136853)
Supplement: Supplementary file 1 [file ijms-22-06853-s001.zip › Supplementary File-Table S2-CHK.pdf]

Table S2. Electrophysiological properties of neurons in response to cytokines and ATP in Figure 5.

Figure 5	concentration	N	Vm (mV)	Rheobase (pA)	Cm (pF)	Rm (mΩ)
**A. Con**		29	-70.84 ± 1.22	38.97 ± 2.5	64.62 ± 3.48	271.91 ± 12.61
**3 dpi**		16	-69.97 ± 2.18	25.63 ± 2.41, **	46.49 ± 4.09, **	334.27 ± 39.11
**TNFα**	10 ng/ml	11	-67.83 ± 2.79	42.73 ± 4.28, ###	50.57 ± 3.89, *	271.88 ± 21.87
**IL1β**	10 ng/ml	11	-64.06 ± 1.09 **	41.82 ± 3.25, ###	59.03 ± 4.52	308.36 ± 23.09
**IL10**	10 ng/ml	10	-71.58 ± 1.72	75 ± 7.19, ***, ###	92.79 ± 8.4, ***, ###	174 ± 18.14, ***, ###
**ATP**	1 mM	14	-75.93 ± 1.41, *	43.57 ± 4.87	82.44 ± 5.39	207.95 ± 20.23
**ATP-γ-S**	100 μM	4	-77.88 ± 1.98, *	45 ± 9.57	89.26 ± 14.8	220.12 ± 44.67
**IL4**	10 ng/ml	16	-66.86 ± 1.09	60 ± 6.89, **, ###	85.81 ± 7.77 ** ###	205.18 ± 21.07, **, ##
**C. 3 dpi + IL10**	10 ng/ml	7	-67.69 ± 2.36	42.86 ± 5.65, ##	54.31 ± 3.32	221.91 ± 20.33 #
**3 dpi + IL4**	10 ng/ml	11	-64.58 ± 1.52, *	43.64 ± 2.44, ###	60.12 ± 5.84	202.57 ± 17.5, ##

Data are mean ± SEM. Asterisks (*) mark significance compared to control group and hashtags (#) mark significance compared to 3 dpi group. */# $p < 0.05$, **/## $p < 0.01$, ***/### $p < 0.001$.
