# Supplementary material for: Roles of Cytokines in the Temporal Changes of Microglial Membrane Currents and Neuronal Excitability and Synaptic Efficacy in ATP-Induced Cortical Injury Model
Source: Int J Mol Sci. 2021 Jun 25;22(13):6853. doi: 10.3390/ijms22136853 (PMC8268462; doi:10.3390/ijms22136853)
Supplement: Supplementary file 1 [file ijms-22-06853-s001.zip › Supplementary File-Table S3-CHK.pdf]

Table S3. Electrophysiological properties of microglia in response to cytokines and ATP in Figure 6.

Figure 6	Concentration	N	Vm (mV)	Vrev (mV)	Cm (pF)	Rm (GΩ)
**A. Con**	-	28	-46.18 ± 3.41	-82.02 ± 1.33	25.44 ± 1.43	3.03 ± 0.24
**TNFα**	10 ng/ml	8	-30.43 ± 3.68, *	-89.85 ± 5.83	24.76 ± 1.21	1.4 ± 0.18, ***
**IL1β**	10 ng/ml	7	-54.1 ± 7.97	-83.74 ± 1.85	30.69 ± 2.24	3.79 ± 0.91
**IL10**	10 ng/ml	7	-51.89 ± 4.02	-81.9 ± 1.94	21.8 ± 1.23	3 ± 0.46
**IL4**	10 ng/ml	9	-45.87 ± 4.94	-99.34 ± 4.53, **	26.23 ± 1.82	5.98 ± 1.99
**ATP-γ-S**	100μM	7	-48.49 ± 7.44	-88.25 ± 1.59, ***	20.44 ± 1.18, *	3.5 ± 0.51
**ATP-γ-S**	300μM	14	-37.23 ± 3.46	-91.25 ± 4.9, **	23.07 ± 0.91	2.42 ± 0.42
**B. ATP**	1 mM	14	-40.52 ± 3.28	-72.21 ± 2.34	22.4 ± 1.7	2.56 ± 0.33
**TNFα**	30 ng/ml	9	-48.54 ± 4.4	-79.64 ± 2.59	22.35 ± 1.43	2.7 ± 0.35
**IL1β**	30 ng/ml	11	-38.34 ± 4.84	-98.86 ± 6.34, *	38.08 ± 6.08	3.82 ± 0.33
**IL10**	30 ng/ml	8	-47.76 ± 6.49	-82.19 ± 1.18	26.14 ± 1.43	3.38 ± 0.39
**IL4**	30 ng/ml	11	-47.46 ± 4.96	-80.74 ± 3.43	20.81 ± 1.54	3.47 ± 0.37
**C. TNFα + IL1β**	Each 10 ng/ml -	12	-37.93 ± 4.68	-78.8 ± 1.93	21.75 ± 2.31	2.88 ± 0.51
**IL10 + IL4**	Each 10 ng/ml -	12	-47.25 ± 5.13	-81.91 ± 1.2	28.35 ± 1.31, *	3.23 ± 0.41
**TNFα + IL1β + IL10 + IL4**	Each 10 ng/ml -	13	-51.44 ± 5.16	-80.19 ± 0.88	27.83 ± 1.26, *	3.07 ± 0.35
**D. 3 dpi**	-	31	-53.13 ± 3.15	-74.58 ± 0.49, ***	30.75 ± 2.24, *	1.16 ± 0.1, ***

**3 dpi + IL10**	10 ng/ml -	8	-68.18 ± 5.23, **, #	-89.58 ± 4.24, *, ##	29.45 ± 1.25, *	2.64 ± 0.57, #
**3 dpi + IL4**	10 ng/ml -	14	-56.06 ± 6.64	-82.36 ± 2.29, ##	41.29 ± 1.89, ***, ###	1.19 ± 0.16, ***
**3 dpi + IL10 + IL4**	10 ng/ml -	12	-69.71 ± 3.97, ***, ##	-73.92 ± 2.57, **	41.03 ± 7.99	1.49 ± 0.23, **

Data are mean ± SEM. Asterisks (*) mark significance compared to control group and hashtags (#) mark significance compared to 3 dpi group. */# $p < 0.05$, **/## $p < 0.01$, ***/### $p < 0.001$.
